# The impact of vitamin D3 supplementation on the faecal and oral microbiome of dairy calves indoors or at pasture

**DOI:** 10.1038/s41598-023-34840-2

**Published:** 2023-06-05

**Authors:** S. Vigors, S. Flores-Villalva, K. G. Meade

**Affiliations:** 1 School of Agriculture and Food Science, University College Dublin, Belfield, Dublin 4, Ireland; 2 CENID Fisiología, INIFAP, Querétaro, México; 3 Conway Institute of Biomolecular and Biomedical Research, University College Dublin, Belfield, Dublin 4, Ireland; 4 Institute of Food and Health, University College Dublin, Belfield, Dublin 4, Ireland

**Keywords:** Microbiology, Animal physiology

## Abstract

Vitamin D (VitD) is emerging as an immune regulator in addition to its established role in metabolism and mineral homeostasis. This study sought to determine if in vivo VitD modulated the oral and faecal microbiome in Holstein–Friesian dairy calves. The experimental model consisted of two control groups (Ctl-In, Ctl-Out) which were fed with a diet containing 6000 IU/Kg of VitD_3_ in milk replacer and 2000 IU/Kg in feed, and two treatment groups (VitD-In, VitD-Out) with 10,000 IU/Kg of VitD_3_ in milk replacer and 4000 IU/Kg in feed. One control and one treatment group were moved outdoors post-weaning at approximately 10 weeks of age. Saliva and faecal samples were collected after 7 months of supplementation and analysis of the microbiome was performed using 16S rRNA sequencing. Bray–Curtis dissimilarity analysis identified that both sampling site (oral vs. faecal) and housing (indoor vs. outdoor) had significant influences on the composition of the microbiome. The calves housed outdoors had greater microbial diversity in the faecal samples based on Observed, Chao1, Shannon, Simpson and Fisher measures in comparison to calves housed indoors (*P* < 0.05). A significant interaction between housing and treatment was observed for the genera *Oscillospira, Ruminococcus*, *CF231* and *Paludibacter* in faecal samples. The genera *Oscillospira* and *Dorea* were increased while *Clostridium* and *Blautia* were decreased following VitD supplementation in the faecal samples (*P* < 0.05). An interaction between VitD supplementation and housing was detected in the abundance of the genera *Actinobacillus* and *Streptococcus* in the oral samples. VitD supplementation increased the genera *Oscillospira*, *Helcococcus* and reduced the genera *Actinobacillus*, *Ruminococcus*, *Moraxella*, *Clostridium*, *Prevotella*, *Succinivibrio* and *Parvimonas*. These preliminary data suggest that VitD supplementation alters both the oral and faecal microbiome. Further research will now be conducted to establish the significance of microbial alterations for animal health and performance.

## Introduction

Infectious diseases significantly impact the economic sustainability of dairy systems, and annual early life mortality can account for almost 10% of calves on average, with rates significantly higher on some farm enterprises^1^. In addition, disease compromises the ability of additional calves to meet production targets and reach their genetic potential. Respiratory and enteric bacteria and viruses (respiratory syncytial virus, BVD, herpesvirus, *E. coli,* rotavirus, *Salmonella*) account for the majority of infectious diseases that affect young dairy calves^2,3^. A maladaptive start in life can continue to compromise the productivity of cattle as well as contribute to disease susceptibility later in life^2^. Therefore, to more adequately address the welfare needs of dairy calves in particular, and to reduce our dependence on antibiotics as a treatment for bacterial infections, continual efforts are required to optimally bolster natural disease resistance and health of the animal.

During early life, calves rely predominantly on their innate immune system for protection against disease as the adaptive arm of their immune system develops gradually to maturity at approximately 6 months after birth^4^. A key contributor to optimal adaptive immune system priming and the development of homeostasis is the establishment of the microbiome. Initial starter cultures for microbial development are thought to originate from colostrum ingested immediately after birth, although more recent research suggests that some exposure may occur in utero^5^. The neonate consumes an exclusively milk diet and it is thought that colonisation of the intestine begins in the ileum and subsequently becomes established throughout the extensive developing pre-ruminant tract^6,7^. The composition of the intestinal microflora has been established in young calves, and a predominance of Firmicutes reported. However considerable dynamic changes occur in microbial succession as the rumen develops and tissue homeostatic regulatory mechanisms occur^8^.

Supporting the establishment of a diverse microbiome is now considered to be a key feature in encouraging optimal immune system development and the optimal establishment of homeostasis. In fact, a diverse microbiome is thought to be a principal protective feature against invading pathogens^9^, and factors which contribute to polarisation or a reduction in diversity of the microbiome (referred to as dysbiosis) are a significant disease risk factor. A recent study in humans has shown that vitamin D (VitD) supplementation significantly increased gut microbial diversity. Specifically, the Bacteroidetes to Firmicutes ratio increased, along with the abundance of the health-promoting probiotic taxa *Akkermansia* and *Bifdobacterium*^10^. The oral microbiome has received less research attention than the intestinal microbiome but studies have identified an abundance of families including *Neisseriaceae*, *Streptococcaceae* and *Pasteurellaceae* as well as *Moraxellaceae* in the oral microbiota^11^, but the effects of VitD on the relative abundance of these populations remains unknown. VitD supplementation may offer a mechanism to support immune system development in young dairy calves, however the role of VitD in the regulation of the microbiome in cattle has not been previously studied.

VitD is an essential steroid for life in most mammals. The two most prominent forms of VitD are ergocalciferol (Vit D_2_) and cholecalciferol (Vit D_3_). Ergocalciferol is derived from the plant steroid, ergosterol, whereas cholecalciferol is produced in the skin following exposure to UVB rays from the sun^12^. Cattle can obtain VitD through dermal synthesis and dietary sources (hay, silage, milk replacers, etc.). The content of Vit D_2_ in silage is often highly variable, and green grass is a poor source of Vit D_2_; thus, for grazing ruminants, almost all VitD comes from dermal synthesis. Although, Vit D_3_ is provided in feed concentrates, differences in husbandry practices causes variation in the VitD status of cattle between farms and throughout the year^13^.

Our work has recently established that circulating levels of VitD are sub-optimal in Spring-born dairy calves^14^, and that low levels are associated with significant temporal changes in systemic immunity including immune cell populations and the expression of immune proteins, including the chemokine, Interleukin 8^15^. Therefore, we hypothesised that supplementation with this micronutrient would significantly alter the oral and faecal microbiome of Holstein–Friesian dairy calves. Utilising the same VitD supplementation model, the aim of this study was to characterise the microbial populations present in the oral cavity and in faeces which may be altered in response to dietary VitD supplementation.

## Results

### Calf vitamin D concentrations and growth performance

**Figure 1:**
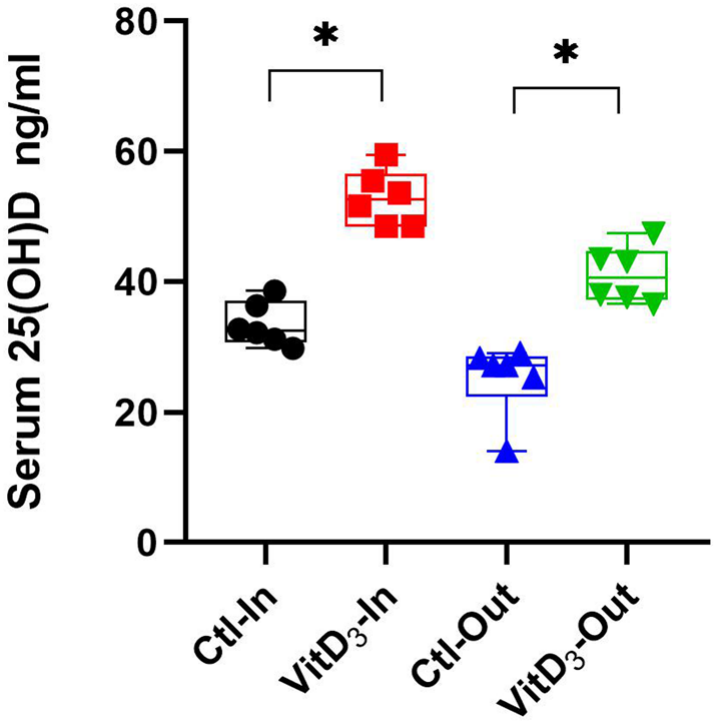
Circulating concentrations of 25OHD levels in serum from Holstein–Friesian calves in each group. Boxplots and individual calf concentrations of serum 25OHD levels in calves (ng/ml) within each group (n = 6) are shown with statistical significance denoted as *p < 0.05. Calves were a subset from a larger study as previously published Flores-Villalva, et al.^14^.

Circulating concentrations of VitD were significantly divergent between groups used for microbiome sequencing (*P* < 0.05, Fig. 1). To ensure no negative impacts on animal growth or welfare these parameters were monitored throughout the experiment. There was no significant difference between treatment groups in relation to incidence of illness (*P* > 0.05). Although the Ctl-Out group showed a tendency toward lower final weights at the end of the experiment, no significant differences in either initial weight or body weight gain was detected (*P* > 0.05; Supplementary Figure S1).

### Distinct difference between faecal and oral microbiome

**Figure 2:**
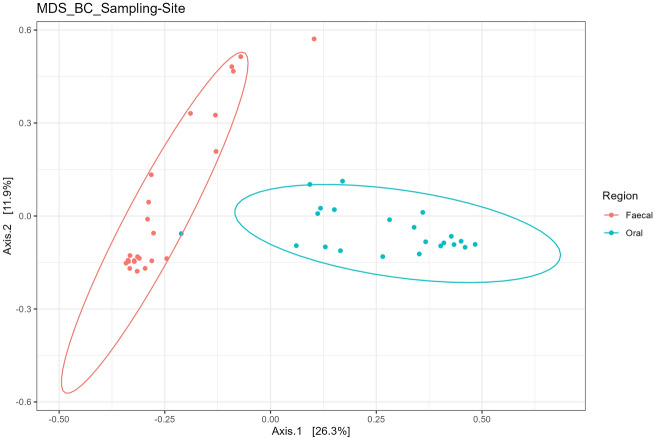
Principal component (PC) analysis based on multiple dimensional scaling and Bray–Curtis distance matrix. The analysis emphasises the clear distinction between faecal and oral samples in Holstein–Friesian calves. N = 22 calves per group.

**Figure 3:**
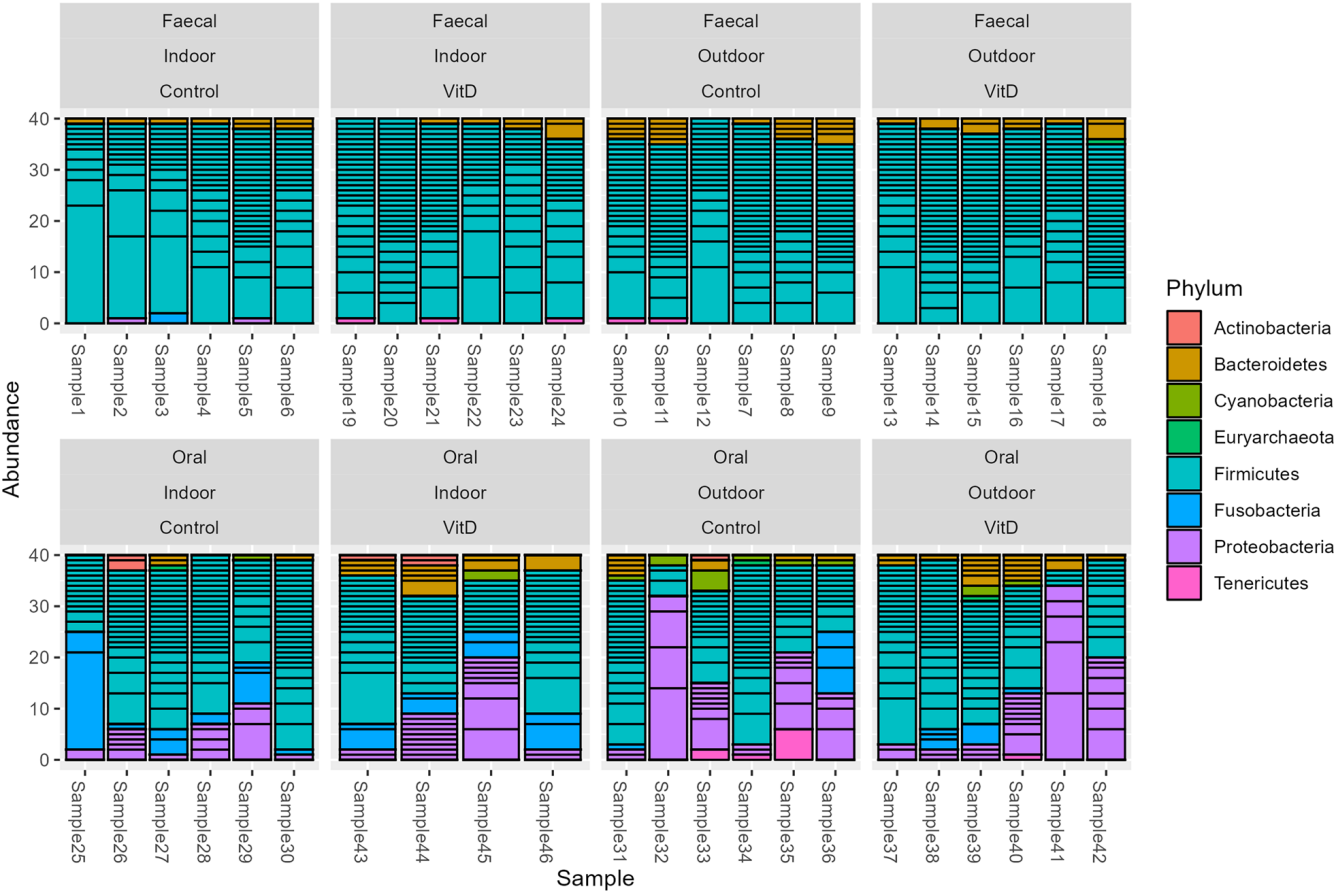
Composition of faecal and oral microbiome at phylum level grouped by treatment and housing. These data illustrate the difference between the oral and faecal microbiome in Holstein–Friesian calves. N = 4–6 calves per group.

The calf faecal and oral microbiome differed significantly (*P* < 0.05) based on Permanova analysis and through visualisation using the Bray Curtis distance matrix and multi-dimensional scaling (Fig. 2). In relation to alpha diversity, the faecal samples were more diverse based on Observed, Chao1 and Shannon measures when compared to oral samples (*P* < 0.05) (data not shown). The difference in composition was further emphasised by the differences at phylum level (Fig. 3). The faecal microbiome had significantly increased Firmicutes (94% vs. 56%) but lower Fusobacteria (< 1% vs. 9%) and Proteobacteria (< 1% vs. 23%) compared to the oral microbiome.

### Impact of VitD and housing on the faecal microbiome

The faecal microbiome was dominated by the family *Ruminococcaceae* (> 70% on average) followed by *Lachnospiraceae* (15%) *Clostridiaceae* (3%), *Peptostreptococcaceae* (2%) and *Rikenellaceae* (1%) with the remainder of families being observed at less than 1% (Supplementary Table S1). At genus level *Oscillospira* (30% on average) was the dominant genera with *Faecalibacterium* (11%), *Dorea* (10%), *Ruminococcus* (6%), *Prevotella* (5%), *CF231* (5%), *Clostridium* (3%) and *Blautia* (2%) being prominent (Supplementary Table S1).

**Figure 4:**
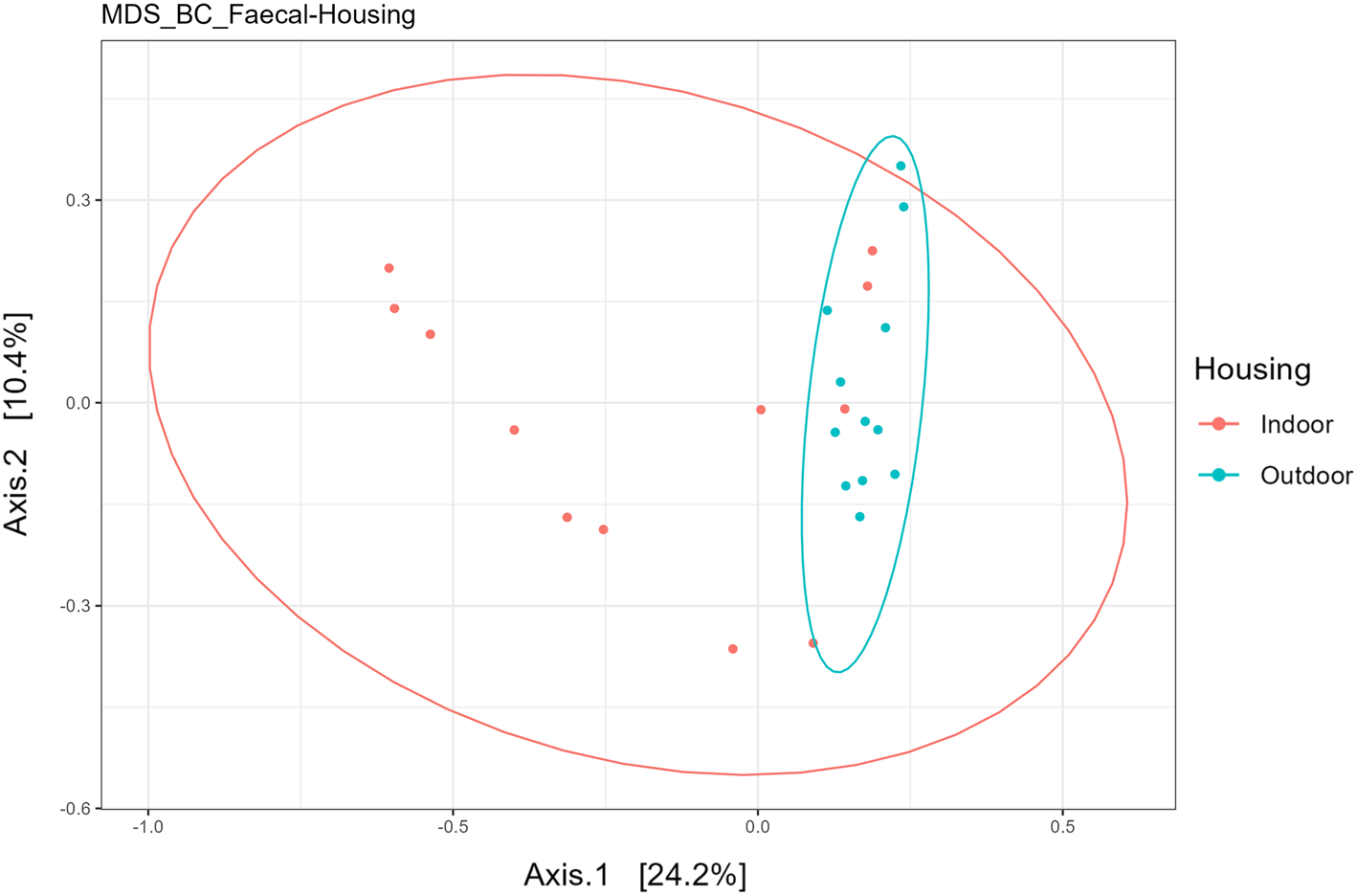
Principal component analysis (PCA) based on multiple dimensional scaling and Bray–Curtis distance matrix. The analysis illustrates the difference between indoor and outdoor housed calves in samples collected from faeces from Holstein–Friesian calves. N = 12 calves per group.

VitD did not impact any measures of alpha diversity in the current study (Table 1). However, housing significantly impacted Beta diversity in the faecal microbiome (*P* < 0.05) based on Permanova analysis and through visualisation using the Bray Curtis distance matrix and multi-dimensional scaling (Fig. 4). The calves housed outdoors had greater diversity based on Observed, Chao1, Shannon, Simpson and Fisher measures in comparison to calves housed indoors (Table 1;* P* < 0.05).

**Table 1:** An assessment of the impact of VitD supplementation on Alpha Diversity in faecal samples in Holstein–Friesian calves.

	Indoor		Outdoor		SEM	Trt^1^	Housing^1^	Trt* Housing^2^
	Control	VitD	Control	VitD				
Faecal								
Observed	162.00	272.83	195.33	259.00	21.49	0.655	0.001	0.286
Chao1	242.13	409.22	294.75	398.53	26.44	0.437	0.001	0.245
Shannon	3.28	4.24	3.91	4.27	0.18	0.078	0.002	0.115
Simpson	0.88	0.95	0.94	0.95	0.02	0.073	0.022	0.055
Fisher	45.35	87.50	62.19	86.80	6.74	0.245	0.001	0.208

^1^Main effects of treatment and housing, ^2^Interaction between treatment and housing.

Significant effects on differential abundance analysis in the faecal samples are presented in Table 2, while full analysis results are presented in Supplementary Table S1. There was no significant impact of VitD supplementation, or no interaction between VitD supplementation and housing type on the faecal microbiome at phylum or family level (Supplementary Table S1). The families *Peptostreptococcaceae*, *Rikenellaceae* and *Ruminococcaceae* were increased in calves kept outdoors while the indoor housed calves had increased *Lachnospiraceae* and *Prevotellaceae* (Table 2; *P* < 0.05).

**Table 2:** Differential abundance analysis of the impact of housing and VitD supplementation on the faecal microbiome in Holstein–Friesian calves.

	Indoor		Outdoor		SEM	Trt^1^	Housing^1^	Trt* Housing^2^
	Control	VitD	Control	VitD				
Phylum								
Firmicutes	94.27	93.37	93.06	94.91	0.44	0.907	0.972	0.732
Bacteroidetes	4.37	5.11	6.37	4.56	0.46	0.63	0.481	0.194
Tenericutes	0.31	0.89	0.41	0.46	0.07	0.337	0.782	0.438
Proteobacteria	0.71	0.56	0.07	0.08	0.10	0.943	0.071	0.877
Family								
*Peptostreptococcaceae*	0.77	0.85	3.69	2.03	0.36	0.504	0.003	0.357
*Rikenellaceae*	0.54	0.73	2.06	1.76	0.22	0.866	0.016	0.593
*Ruminococcaceae*	68.03	72.41	75.08	79.89	1.74	0.206	0.047	0.999
*Lachnospiraceae*	20.5	18.15	11.87	11.61	1.49	0.509	0.011	0.647
*Prevotellaceae*	3.22	3.26	0.06	0.04	0.58	0.917	0.004	0.911
Genus								
*Ruminococcus*	6.27	4.31	4.63	10.69	1.29	0.193	0.092	0.002
*CF231*	0.08	2.07	12.22	3.82	1.24	0.176	0.001	0.008
*Paludibacter*	0.05	0.86	3.63	0.49	0.57	0.651	0.079	0.027
*Oscillospira*	17.83	27	40.82	44.29	3.30	0.004	0.001	0.041
*Dorea*	6.9	11.48	9.23	14.34	1.49	0.002	0.063	0.796
*Clostridium*	4.63	1.68	4.01	3.63	1.37	0.048	0.248	0.096
*Blautia*	3.69	3.07	1.62	0.32	0.56	0.048	0.002	0.109
*Prevotella*	10.09	8.91	0.31	0.28	1.57	0.822	0.001	0.999
*5-7N15*	0.37	0.85	1.98	2.01	0.28	0.357	0.011	0.368

^1^Main effects of treatment and housing, ^2^Interaction between treatment and housing.

There was an interaction between housing and treatment for the genera *Ruminoccocus*, *CF231 Paludibacter and Oscillospira* (Table 2; *P* < 0.05). There was an interaction between housing and treatment for the genera *Ruminococcus* with VitD supplementation increasing *Ruminococcus* outdoors while no effect was identified for indoor housed calves. There was an interaction between housing and treatment for the genus *CF231* with VitD supplementation increasing *CF231* in the indoor housing while VitD supplementation decreased this genus when calves were outdoors. There was an interaction between housing and treatment for the genus *Paludibacter* with no effect identified in the indoor housed calves while VitD supplementation outdoors significantly reduced *Paludibacter*. There was a greater response to VitD supplementation for the genus *Oscillospira* for indoor housed compared to calves outdoors where supplementation had no effect. Irrespective of housing type the genera *Dorea* was increased while *Clostridium* and *Blautia* were decreased following VitD supplementation (*P* < 0.05). Calves kept indoors had increased *Faecalibacterium*, *Blautia*, *Prevotella* and *Succinivibrio* while *5-7N15* was reduced compared to calves housed outdoors (*P* < 0.05).

### Impact of VitD and housing on the oral microbiome

The oral microbiome was compromised of *Ruminococcaceae* (32%) *Pasteurellaceae* (17%), *Lachnospiraceae* (10%), *Fusobacteriaceae* (6%), *Leptotrichiaceae* (5%), *Neisseriaceae* (3%), *Moraxellaceae* (2%), *Peptostreptococcaceae* (2%), *Clostridiaceae* (2%), *Tissierellaceae* (2%), *Flavobacteriaceae* (2%), *Mycoplasmataceae* (2%) and *Weeksellaceae* (2%) (average values from data shown in Supplementary Table S3). At genus level the oral microbiome was compromised of *Actinobacillus* (19%), *Fusobacterium* (12%), *Moraxella* (5%), *Ruminococcus* (4%), *Faecalbacterium* (4%), *Clostridium* (4%), *Oscillospira* (4%), *Dorea* (3%), *Ornithobacterium* (3%), *Helococcus* (2%) and *Blautia* (1%) with other genera identified at less than 1% abundance.

The measures of diversity were not impacted in the oral samples (Supplementary Table S2). Significant effects on differential abundance analysis in the oral samples are presented in Table 3, while full analysis results are presented in Supplementary Table S3. There was an interaction between treatment and housing for the phyla Firmicutes and Proteobacteria (*P* < 0.05). VitD supplementation decreased Firmicutes in indoor housed calves while Firmicutes was increased when calves were outdoors. There was an interaction between treatment and housing for the phylum Proteobacteria with VitD supplementation increasing Proteobacteria indoors while no effect was identified outdoors. Calves supplemented with VitD had increased Bacteroidetes compared to calves who were not fed additional VitD (*P* < 0.05). The phyla Fusobacteria, and Actinobacteria were reduced while Tenericutes and Cyanobacteria were increased in calves outdoors compared to calves housed indoors (Table 3).

**Table 3:** Differential abundance analysis of the impact of housing and VitD supplementation on the oral microbiome in Holstein–Friesian calves.

	Indoor		Outdoor		SEM	Trt^1^	Housing^1^	Trt* Housing^2^
	Control	VitD	Control	VitD				
Phylum								
Firmicutes	66.2	53.55	50.53	54.55	4.87	0.259	0.044	0.023
Proteobacteria	10.29	20.53	29.45	31.56	4.86	0.001	0.001	0.006
Fusobacteria	15.39	12.11	5.43	3.54	2.52	0.059	0.001	0.581
Bacteroidetes	5.38	9.98	5.98	6.01	0.86	0.079	0.245	0.083
Actinobacteria	1.8	2.34	0.61	0.36	0.28	0.776	0.007	0.421
Tenericutes	0.32	0.26	3.93	1.69	0.78	0.412	0.003	0.633
Cyanobacteria	0.27	0.61	4.01	2.02	0.82	0.915	0.002	0.184
Family								
*Pasteurellaceae*	2.12	15.65	24.89	29.02	5.14	0.001	0.001	0.001
*Lachnospiraceae*	14.24	7.69	8.65	9.58	1.09	0.087	0.339	0.021
*Streptococcaceae*	0.22	3.55	0.48	0.05	0.55	0.853	0.119	0.031
*Neisseriaceae*	3.88	3.68	3.6	0.87	0.94	0.023	0.021	0.033
*Flavobacteriaceae*	1.94	4.26	1.22	4.39	0.58	0.002	0.452	0.393
*Moraxellaceae*	2.95	0.63	3.36	2.42	0.73	0.024	0.067	0.126
*Leptotrichiaceae*	9.95	6.35	1.39	0.74	2.03	0.106	0.001	0.778
*Weeksellaceae*	2.31	5.53	0.47	0.8	0.55	0.106	0.001	0.686
*Fusobacteriaceae*	7.01	7.12	4.63	3.63	1.13	0.554	0.011	0.501
*Mycoplasmataceae*	0.31	0.21	4.14	1.85	0.91	0.385	0.003	0.782
*Corynebacteriaceae*	1.11	1.55	0.06	0.19	0.05	0.472	0.021	0.702
Genus								
*Actinobacillus*	2.93	17.47	21.91	27.51	5.04	0.001	0.001	0.001
*Streptococcus*	0.73	6.87	1.11	0.11	1.06	0.988	0.015	0.004
*Dorea*	4.61	2.09	2.12	4.01	0.66	0.778	0.812	0.014
*Ruminococcus*	11.99	2.25	3.61	1.51	1.21	0.001	0.007	0.151
*Moraxella*	4.54	1.01	9.03	5.92	2.09	0.004	0.001	0.075
*Flavobacterium*	0.05	0.95	0.61	6.64	1.26	0.014	0.036	0.794
*Succinivibrio*	0.88	0.31	2.39	0.23	0.38	0.021	0.607	0.348
*Prevotella*	2.36	0.89	1.14	0.39	0.30	0.046	0.123	0.922
*Clostridium*	4.63	1.68	4.01	3.63	0.86	0.048	0.248	0.096
*Sphingomonas*	0.91	0.89	2.31	4.71	0.15	0.372	0.003	0.345
*Oscillospira*	2.02	2.84	4.03	6.88	1.31	0.089	0.005	0.694
*Fusobacterium*	18.19	16.35	8.99	8.45	2.56	0.509	0.001	0.861
*Ornithobacterium*	4.41	7.51	1.16	1.04	0.85	0.497	0.001	0.311

^1^Main effects of treatment and housing, ^2^Interaction between treatment and housing.

There was an interaction between treatment and housing on the families *Pasteurellaceae*, *Lachnospiraceae, Streptococcaceae* and *Neisseriaceae* (Table 3). VitD supplementation indoors increased *Pasteurellaceae* to a greater extent than calves outdoors. VitD supplementation indoors decreased *Lachnospiraceae* while no effect was identified outdoors. There was an interaction between VitD supplementation and housing on the family *Streptococcaceae* which was increased in the indoor housed calves and reduced in the calves outdoors. VitD supplementation decreased the family *Neisseriaceae* in the calves outdoors while this genus was unaffected when calves were indoors. VitD supplementation increased the family *Flavobacteriaceae* while *Leptotrichiaceae* and *Moraxellaceae* were lower in calves that did not receive VitD (*P* < 0.05; Table 3). When comparing calves housed outdoors vs calves housed indoors *Leptotrichiaceae*, *Fusobacteriaceae*, *Weeksellaceae*, *Corynebacteriaceae* were higher in indoor housed calves while *Mycoplasmataceae* was reduced when compared to calves in the VitD outdoor group (*P* < 0.05; Table 3).

There was an interaction between VitD supplementation and housing in the abundance of the genera *Actinobacillus*, *Streptococcus* and *Dorea* (*P* < 0.05; Table 3). Supplementation indoors increased *Actinobacillus* to a greater extent than when calves were outdoors (*P* < 0.05). The interaction between VitD supplementation and housing for the genus *Streptococcus* indicated that supplementation increased *Streptococcus* in the indoor housing while there was no effect when calves were outdoors. In the case of *Dorea,* VitD supplementation reduced the genus indoors while it was increased in calves outdoors (*P* < 0.05). VitD supplementation reduced the genera *Ruminococcus*, *Moraxella*, *Succinivibrio, Prevotella* and *Clostridium* while *Flavobacterium* was increased irrespective of housing type (*P* < 0.05; Table 3). When comparing outdoor versus indoor housed calves, calves outdoors had higher *Moraxella*, *Flavobacterium, Sphingomonas, Oscillospira* while calves indoors had higher *Streptococcus, Fusobacterium*, *Ruminococcus* and *Ornithobacterium* (P < 0.05; Table 3).

## Discussion

Adequate VitD is now viewed as vital for optimal health with research identifying important associations between 25OHD blood concentration and immune function. VitD acts as an activator of the innate immune system to enhance the response to infection. VitD stimulates the microbicidal capacity of the innate cells against pathogens by promoting the expression of antimicrobial proteins, lowering the intracellular ion concentration and enhancing autophagy^16,17^. We have recently shown how exogenous VitD promotes mycobacterial killing in peripheral blood cells from calves^18^. Furthermore, VitD regulates the activity of the adaptive immune system, with effects commonly described as an inhibition of the Th1 response and promotion of Th2 cell differentiation. Therefore, VitD limits the development of an excessive inflammatory response^19^. Circulating VitD levels in Spring-born dairy calves are deficient at birth and sub-optimal up until approximately 5 months of life^14,20^. Increasingly associations are being established between the microbiome and host health in calves^21^ and due to the intrinsic link between immune function and the microbiome, the influence of VitD supplementation on the microbiome of the calf warrants further exploration. Our previous work formed an important baseline in the characterisation of the age-related development of the microbiome^8^ and here we identify the diversity and compositional changes associated with VitD supplementation. The association between VitD and the microbiome has been the subject of multiple recent studies (as reviewed by Tangestani et al.^22^). Although the mechanisms of the effects are the subject of on-going analyses, it has been suggested that VitD may upregulate host defence peptide expression in the intestine and alter the expression of tight junction proteins via modulation of VDR which is widely expressed in intestinal tissues^10^. Therefore, the aim of this study was to characterise the microbial populations present in the oral cavity and in faeces which may be altered in response to dietary VitD supplementation across two different housing conditions.

In this study, Firmicutes represented over 90% of the faecal microbiome whilst the oral microbiome was comprised of ~ 55% Firmicutes, ~ 20% Proteobacteria, ~ 10% Fusobacteria and ~ 5% Bacteroidetes. This agreed in part with previous findings where Firmicutes was the dominant phyla in faecal samples of calves^8,11^. In the study of Klein-Jöbstl, et al.^23^ *Ruminococcaceae* was the dominant family in the faecal microbiome of cows which is in agreement with the current study. However, in the study of Barden, et al.^11^ Proteobacteria was the most abundant phyla in the oral samples whereas here Firmicutes was most abundant. Similarities also exist between the current dataset and previous studies as families including *Pasteurellaceae* and *Moraxellaceae* were also highly abundant in calf oral samples^11^. In the study of Owens, et al.^24^ Proteobacteria dominated the oral microbiome whereas Bacteroidetes was dominant in the faecal microbiome of calves. The age of calves is likely a significant factor contributing to the divergence between reports in the literature with sampling varying from 28 days of age^11^, to 60 days of age^24^ and the current study where calves were 230 days old.

There were several interesting changes associated with VitD on the faecal microbiome and in particular effects that differed across housing type. There was a greater response to VitD supplementation for the genus *Oscillospira* for indoor housed calves compared to outdoor housed calves where supplementation had no effect. The opposite trend was identified for *Ruminococcus* with VitD supplementation having an effect when calves were outdoors but not housed indoors. *Oscillospira* degrades plant cell walls and is increased when feeding fresh forage^25–27^. Previously, *Oscillospira* was shown to be more abundant in steers grazing pasture in comparison to steers indoors^27^. When the diet was changed from pasture to indoor feeding *Oscillospira* abundance diminished emphasising a clear link with pasture grazing^27^. This then explains the difference between indoor and outdoor housed calves in relation to the microbiome which is likely linked to differences in the diet offered. In the case of *Ruminococcus*, this genus was not impacted indoors but was increased by over 6% outdoors following VitD supplementation. *Ruminococcus* has similar functionality to *Oscillospira* and is involved in the degradation of forages. Within the Firmicutes Phylum and the *Lachnospiraceae* family, *Dorea* was increased irrespective of housing type while *Blautia* was reduced. *Dorea* is active in cellulose and hemicellulose degradation and has been attributed with improved gut health in neonatal ruminants and increased ADG^28–30^. *Blautia* is correlated with propionate production following starch fermentation and has also been associated with improved ADG in calves^31,32^. The genus *Clostridium* was also reduced following VitD supplementation. *Clostridium* contains species that cause disease in calves in particular *C.perfringens* and *C.difficile*^33^. These changes suggest overall positive impacts on the intestinal microbiome following VitD supplementation.

Taxonomic differences are associated with VitD supplementation in this study but the reason for the interaction between VitD and the microbiome is still unclear. While still not fully confirmed it seems that the effects on the microbiome in this study are not a direct influence but rather through action following absorption of the micronutrient. VitD was previously established to be stable in the rumen with no degradation detected, and with absorption taking place in the small intestine in previous studies^34^. Furthermore, VitD did not impact rumen fermentation parameters in an in vitro cow study^35^. This suggests that the influence of VitD on the microbiome is potentially occurring through modification of the host, likely through the interaction between the microbiome and the host at the intestinal epithelium. This influence of VitD on the host, in particular host immunity was emphasised with a study conducted on the same group of calves^14^. Calves supplemented with VitD_3_ indoors had lower circulating neutrophils, eosinophils and basophils compared to control fed calves^14^. Previously, host genetics has been established to influence the microbiome of ruminants as both *Roseburia* and *Oscillospira* were associated with SNPs regulating host immunity and metabolism linking a potential route for the VitD in this study to influence the microbiome^36^. In this study calves were either raised indoors on grass silage or outdoors on pasture. This could explain the differences in the impact of VitD as husbandry practices are known to influence VitD metabolism^37^. Indeed in human and mouse studies a link has been made between VitD and the microbiome^38,39^. Interestingly two families (*Ruminococcaceae* and *Lachnospiraceae*) that were higher in wild type vs mice that cannot produce 1,25(OH)_2_D_3_^40^ were also impacted by VitD in the current study. Further research will need to be conducted to better understand this link, in particular by sampling directly from the various regions of the digestive tract rather than using faecal samples as a proxy.

In the oral samples, within the Proteobacteria phylum there was interaction between VitD supplementation and housing on the genus *Actinobacillus* with VitD increasing this taxon by 15% indoors but only by 6% outdoors. In the same phyla *Moraxella* and *Succinivibrio* were reduced following VitD supplementation irrespective of housing. Several species of *Actinobacillus* are associated with causing the disease Actinobacillosis (wooden tongue) so the increase in this taxon following VitD supplementation is surprising due to the links between VitD with improved animal health. In contrast the genera *Moraxella* and *Succinivibrio* were reduced following VitD supplementation. *Moraxella* is linked to the ocular infections through *Moraxella Bovis*^41^. In the Firmicutes phylum and the Order Clostridia, there were several genera that were influenced by VitD. There was an interaction between housing and VitD on the genus *Dorea* which was reduced in indoor housed calves but increased outdoors which differs from the effect seen in the faeces where supplementation increased the genus *Dorea* both indoors and outdoors. One effect that could be beneficial is the reduction in the genus Clostridium which was reduced following VitD supplementation. *Clostridium* is a genus known to contain a number of species that cause disease in calves in particular *C.perfringens* and *C.difficile*^33^. Interestingly, *Clostridium* was also reduced in the faecal samples. However, in contrast to the reduction of *Clostridium*, the genus *Streptococcus* was increased following the supplementation of VitD. Streptococcus are a genus known to contain a number of pathogenic bacteria such as *Streptococcus bovis*^42^ so again the fact VitD is increasing this genus is surprising. Further research needs to be conducted to examine the influence these identified changes are having on the calf.

While the primary aim of this study as to assess the impact of VitD supplementation in two different housing types (indoors vs outdoors) it is also of interest to compare the microbiome of the indoor and outdoor fed calves irrespective of supplementation. Beta diversity analysis is used to determine compositional dissimilarities between sample types and indicated a clear distinction between housing type in the faecal samples but no difference in the oral samples. There are numerous factors that could in fact be causing this difference in indoor housed versus calves outdoors that could be influencing the microbiome. Indoor versus outdoor groups were similarly managed up until weaning on approximately d70. Following this timepoint the indoor group remained inside and were fed ad libitum on grass silage and hay while the other group were moved outdoors and were offered grazed grass. These dietary differences are likely the dominant factor in the alterations in the microbiome identified. Differences in alpha diversity were identified in the faecal samples with calves housed outdoors having increased diversity based on all measures of diversity examined with no difference in the oral samples. Interestingly this points to substantial changes in taxa abundance, taxa richness and evenness. Increased bacterial diversity is associated with increased robustness against environmental influences^43^ and improved gut health^44^. As the effect of housing was not the primary focus of this study there are several factors that were not controlled such as diet and the environment that could be impacting the microbiome, but a future more detailed study is required to establish the influence of housing type on the calf microbiome. In addition, the final weights from the calves in each group suggests that Ctl-Out calves have a lower bodyweight, although this difference is not significant. It will be of relevance to future studies to examine if VitD supplementation supports calf growth in a larger cohort of calves.

## Conclusions

Neonatal mortality and morbidity remains a significant issue for the dairy industry^3^ and management systems need to adapt to reduce negative welfare and antibiotic-dependent practices. Nutritional strategies to support optimal immune system development may hold significant promise to reduce these losses, particularly in intensively reared dairy calves. Multiple factors are known to regulate VitD_3_ skin synthesis, including latitude, altitude, and time of exposition to sunlight^45^. Here, this preliminary analysis suggests that VitD supplementation significantly alters the microbiome in calves. The antimicrobial and immunoregulatory role of VitD may offer a low-cost and effective supplement to boost immune function in the calf^46^ and the impact on the oral and faecal microbiome in this study offers an intriguing area for further investigation on fermentation, nutrient digestibility and health. These alterations in the microbial populations may help increase disease resilience in dairy calves.

## Methods

### Study population

Animals used in this study were part of the study described by Flores-Villalva, et al.^14^ and is briefly described below. Forty-eight Holstein–Friesian bull calves with an average weight of 42 kg from a single farm, born between February and March 2020 were enrolled in the experiment. The experiment was a randomized complete block design with a two-by-two factorial arrangement of treatments, with calves randomly assigned to one of 4 treatments (n = 12). Treatments were arranged as a factorial design with two vitamin D_3_ diets (Ctl and VitD) and two sunlight access conditions (indoors = In, or outdoors = Out). A commercial milk replacer (MR) with 6000 IU/kg of VitD_3_ and a commercial pellet with 2000 IU/kg of VitD_3_ were used for the Ctl diets. For the VitD_3_ diets, the MR and pellet were supplemented with VitD_3_ to achieve 10,000 IU/kg and 4000 IU/kg of VitD_3_, respectively. Therefore, the four treatments with 12 calves each were: Ctl-In: Indoors and 6000 IU/Kg in MR + 2000 IU/Kg of VitD_3_ in feed; VitD-In: Indoors and 10,000 IU/Kg in MR + 4000 IU/Kg of VitD_3_ in feed; Ctl-Out: Outdoors and 6000 IU/Kg in MR + 2000 IU/Kg of VitD_3_ in feed; VitD-Out: Outdoors and 10,000 IU/Kg in MR + 4000 IU/Kg of VitD_3_ in feed. The milk replacer was fed until weaning following which supplementation was provided in the feed alone. Compositional analysis of both commercial milk replacer and commercial pellets is provided in supplementary data. Post weaning treatments were fed from d70 until d230 at the completion of the trial. Calves were weighed at the start of the experiment and subsequently on day 70, d130, d160 and d230. At the beginning of the experiment a single injection of 50,000 IU of VitD_3_ was administered subcutaneously to all calves, except Ctl-In, which received a vehicle injection with ethanol. The VitD_3_ was prepared from dry powder concentrate (Rovimix D3 500, DSM Nutritional Products) containing 500,000 IU per gram of VitD_3_ by adding 0.5 g of the concentrate to distilled water. The supplements were prepared fresh weekly and stored at 4 °C. Supplements were added once daily to the MR, and top dressed on the pellets after weaning.

All calves were group housed at Teagasc Animal and Bioscience Research Centre, Co. Meath, Ireland. After weaning at 70 days average outdoor groups (Ctl-Out, VitD-Out) were moved to grazing areas and rotationally grazed from May to October. Indoor groups (Ctl-In, VitD-In) were kept in confinement during the duration of the trial and were offered hay and silage ad libitum. Analysis of 25OHD serum concentrations were done as part of a previous study^14^. During the course of the experiment, signs of respiratory or gastrointestinal disease were registered; however, no differences between groups were observed. One calf was lost but it was not related to experimental treatments. Weight of each calf was recorded at birth and at the end of the trial and it is presented in Supplementary Figure S1.

### Sample collection

To examine the impact of VitD_3_ supplementation and housing type on the microbiome, swabs were collected from the buccal region of the mouth and from the rectum after 230 days of supplementation from six calves per treatment group. Swabs were inserted into the mouth or the rectum, rotated clockwise three times and stored into tubes containing PBS buffer and transferred into dry ice. Then, swabs were stored at − 80 °C until analysis.

### DNA extraction and 16 s rRNA sequencing

Extraction of the bacterial DNA from the faecal and oral swabs and subsequent high-throughput sequencing of the V3–V4 hypervariable region of the bacterial 16S rRNA gene was performed on an Illumina MiSeq platform according to their standard protocols (Eurofins Genomics, Ebersberg, Germany) and as previously described^47^. The V3–V4 region was PCR-amplified with universal primers taking in adapters including nucleotide sequences for forward and reverse index primers. Amplicon purification was conducted with AMPure XP beads (Beckman Coulter, Indianapolis, IN, USA) and prepared for index PCR using Nextera XT index primers (Illumina, San Diego, CA, USA)^48^. The purification step was repeated on the indexed samples using AMPure XP beads and assessed using a fragment analyzer (Agilent, Santa Clara, CA, USA). Following this step, a pool was made using equal quantities from each experimental sample. The library was then analysed using the Bioanalyzer 7500 DNA kit (Agilent) and sequenced using the V3–V4 chemistry (2 × 300 bp paired-end reads).

### Bioinformatic & statistical analyses

Quantitative Insights into Microbial Ecology (Qiime) was used to examine the sequencing data^49^. Sequencing primers were removed using the cutadapt package and the resulting paired end reads were merged using the paired-end reads function within Qiime using the standard criteria. Demultiplexing of paired end raw reads was through the split libraries function and quality filtering was conducted utilizing default QIIME parameters. Only reads that contained no ambiguous characters, no non-exact barcode matches, a sequence length > 225 nucleotides and a read-quality score of > 27 were retained. The uclust function in Qiime was used to pick OTUs based on a sequence similarity of 97%. Singletons were removed, as only OTUs that were present at the level of at least two reads in more than one sample were retained while chimeric sequences were removed using ChimeraSlayer^50,51^. The GreenGenes database assigned OTUs to different taxonomic levels. A combination of the mormalized OTU table, experimental phenotypic data and the phylogenetic tree were combined to produce the phyloseq object for further analysis (http://www.r-project.org; version 3.5.0, accessed on 25 March). The dynamics of richness and diversity in the microbiota were computed with the observed, Chao1, Shannon, Simpson and Fisher indices. The Simpson and Shannon indices of diversity account for both richness and evenness parameters. The beta diversity measurements are a measure of separation of the phylogenetic structure of the OTU in one sample compared with all other samples. This was estimated by normalising the data so taxonomic feature counts were comparable across samples. Several distance metrics were considered, in order to calculate the distance matrix of the different multidimensional reduction methods. These included weighted/unweighted UniFrac distance and non-phylogenetic distance metrics (i.e., Bray–Curtis, Jensen–Shannon divergence and Euclidian) using phyloseq in R^52,53^. Differential abundance testing was performed on tables extracted from the phyloseq object at phylum, family and genus level. The data was analysed using the PROC Glimmix procedure within Statistical Analysis Software (SAS) 9.4 (SAS, 2013). The model assessed the main effects of treatment (Ctrl vs. VitD) and housing (Indoor vs. Outdoor) and their associated interaction with the individual calf being the experimental unit. 6 calves per treatment group were used for the statistical analysis of the relative bacterial abundances with the exception of VitD-In group in the oral samples which only contained four samples due to inadequate DNA in the swabs. Results are presented using Benjamini–Hochberg (BH) adjusted *P* values.

### Ethical approval

All experimental procedures were approved by the Teagasc Ethics Committee (TAEC237-2019) and were conducted under the experimental license (AE19132/P105) from the Health Products Regulatory Authority in accordance with the cruelty to Animals Act (Ireland 1876) and the European Community Directive 2010/63/EU. Reporting in the manuscript follows the recommendations in the ARRIVE guidelines.

## Supplementary Information

Supplementary Information.

## Data Availability

The 16S rRNA gene sequence data were deposited in the European Nucleotide Archive (ENA) under the study accession number PRJEB56677.
