# Supplementary material for: The impact of vitamin D3 supplementation on the faecal and oral microbiome of dairy calves indoors or at pasture
Source: Sci Rep. 2023 Jun 5;13:9111. doi: 10.1038/s41598-023-34840-2 (PMC10241916; doi:10.1038/s41598-023-34840-2)
Supplement: Supplementary file 1 — Supplementary Information. [file 41598_2023_34840_MOESM1_ESM.docx]

**
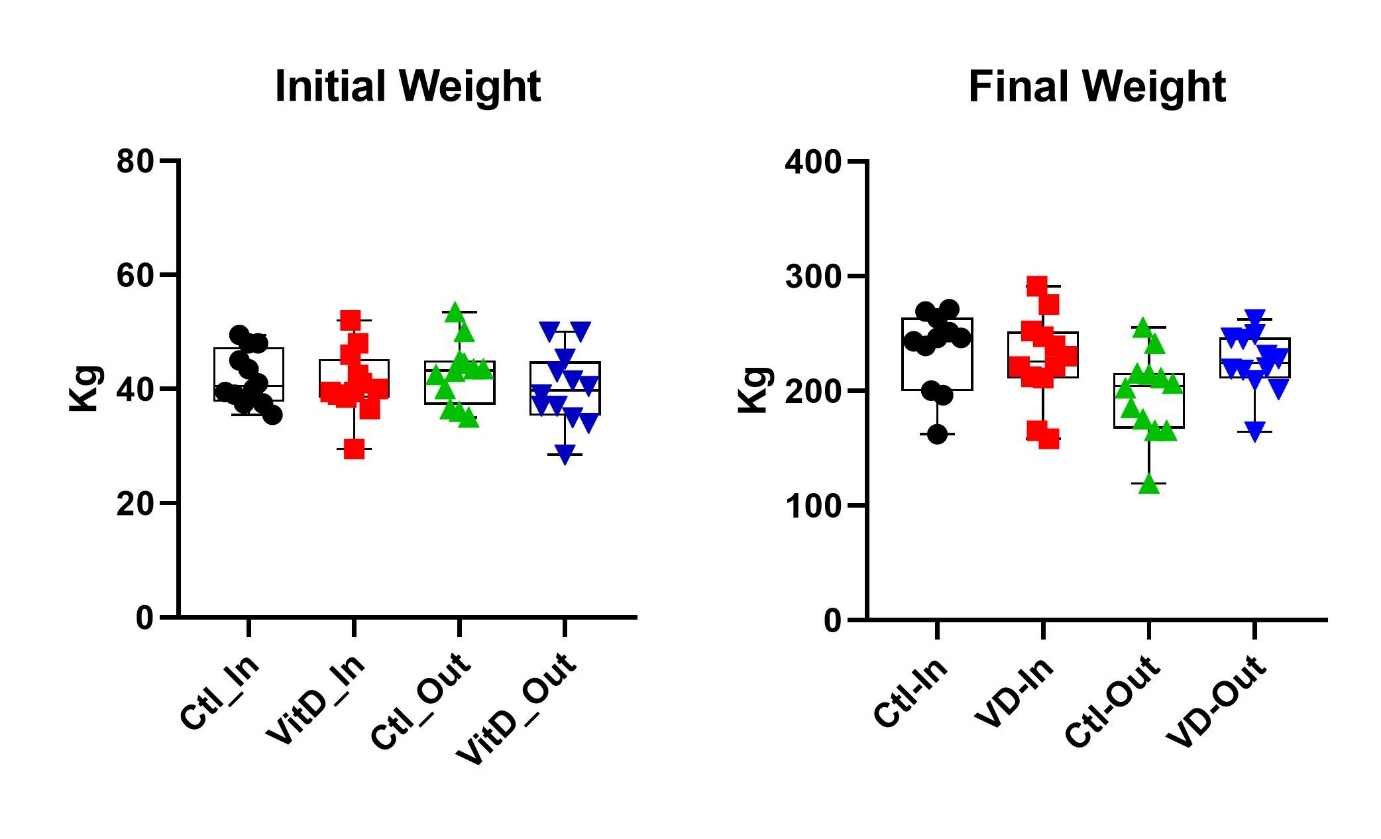
**

**Supplementary Figure S1:** Initial and final weights (Kg) for all calves enrolled in experiment, n=11/12 per group.

**Supplementary Table S1: Differential abundance analysis of the impact of housing and vitamin D supplementation on the faecal microbiome**

|  | Indoor | | Outdoor | |  |  |  |  |
| --- | --- | --- | --- | --- | --- | --- | --- | --- |
|  | Control | VitD | Control | VitD | SEM | Trt^1^ | Housing^1^ | Trt*Housing^2^ |
| **Phylum** |  |  |  |  |  |  |  |  |
| Firmicutes | 94.27 | 93.37 | 93.06 | 94.9 | 0.44 | 0.907 | 0.97 | 0.732 |
| Bacteroidetes | 4.37 | 5.11 | 6.37 | 4.56 | 0.46 | 0.63 | 0.48 | 0.194 |
| Tenericutes | 0.3 | 0.89 | 0.41 | 0.46 | 0.07 | 0.337 | 0.782 | 0.438 |
| Proteobacteria | 0.71 | 0.56 | 0.07 | 0.08 | 0.10 | 0.943 | 0.071 | 0.877 |
| **Family** |  |  |  |  |  |  |  |  |
| *Ruminococcaceae* | 68.03 | 72.41 | 75.08 | 79.89 | 1.74 | 0.206 | 0.047 | 0.999 |
| *Lachnospiraceae* | 20.5 | 18.15 | 11.87 | 11.61 | 1.49 | 0.509 | 0 | 0.647 |
| *Clostridiaceae* | 4.97 | 2.49 | 2.9 | 2.53 | 0.44 | 0.095 | 0.283 | 0.255 |
| *Peptostreptococcaceae* | 0.77 | 0.85 | 3.69 | 2.03 | 0.36 | 0.504 | 0.003 | 0.357 |
| *Rikenellaceae* | 0.54 | 0.73 | 2.06 | 1.76 | 0.22 | 0.866 | 0.016 | 0.593 |
| *Prevotellaceae* | 3.22 | 3.26 | 0.06 | 0.04 | 0.58 | 0.917 | 0.004 | 0.911 |
| *Paraprevotellaceae* | 0.08 | 0.57 | 1.95 | 0.64 | 0.18 | 0.617 | 0.059 | 0.076 |
| *Bacteroidaceae* | 0.28 | 0.24 | 0.46 | 0.44 | 0.06 | 0.896 | 0.465 | 0.934 |
| *S24-7* | 0.09 | 0.16 | 0.58 | 0.06 | 0.06 | 0.509 | 0.691 | 0.265 |
| *Erysipelotrichaceae* | 0.1 | 0.24 | 0.18 | 0.31 | 0.04 | 0.48 | 0.678 | 0.867 |
| *Succinivibrionaceae* | 0.64 | 0.21 | 0.03 | 0.05 | 0.08 | 0.872 | 0.17 | 0.623 |
| *Porphyromonadaceae* | 0.02 | 0.15 | 0.69 | 0.11 | 0.10 | 0.965 | 0.343 | 0.265 |
| *Anaeroplasmataceae* | 0.2 | 0.32 | 0.07 | 0.1 | 0.06 | 0.715 | 0.357 | 0.98 |
| *Planococcaceae* | 0.05 | 0.05 | 0.12 | 0.17 | 0.03 | 0.884 | 0.476 | 0.925 |
| *Mogibacteriaceae* | 0.03 | 0.02 | 0.09 | 0.06 | 0.01 | 0.837 | 0.615 | 0.95 |
| **Genus** |  |  |  |  |  |  |  |  |
| *Oscillospira* | 17.83 | 27 | 40.82 | 44.29 | 3.30 | 0.004 | 0 | 0.041 |
| *Dorea* | 6.9 | 11.48 | 9.23 | 14.34 | 1.49 | 0.002 | 0.063 | 0.796 |
| *Coprococcus* | 5.61 | 7 | 6.44 | 6.64 | 0.62 | 0.445 | 0.792 | 0.56 |
| *Ruminococcus1* | 6.27 | 4.31 | 4.63 | 10.69 | 1.29 | 0.193 | 0.092 | 0.002 |
| *Clostridium* | 4.63 | 1.68 | 4 | 3.63 | 1.37 | 0.048 | 0.248 | 0.096 |
| *Faecalibacterium* | 26.97 | 18.46 | 0.55 | 0.28 | 4.13 | 0.286 | 0 | 0.768 |
| *Ruminococcus2* | 2.3 | 1.68 | 3.91 | 1.6 | 0.89 | 0.044 | 0.402 | 0.316 |
| *Blautia* | 3.69 | 3.07 | 1.62 | 0.32 | 0.56 | 0.048 | 0.002 | 0.109 |
| *Prevotella1* | 10.09 | 8.91 | 0.31 | 0.28 | 1.57 | 0.822 | 0 | 0.999 |
| *Roseburia* | 1.88 | 2.27 | 0.96 | 1.6 | 0.26 | 0.3 | 0.138 | 0.629 |
| *CF231* | 0.08 | 2.07 | 12.22 | 3.82 | 1.24 | 0.176 | 0.001 | 0.008 |
| *5-7N15* | 0.37 | 0.85 | 1.98 | 2 | 0.28 | 0.357 | 0.011 | 0.368 |
| *Anaerostipes* | 0.59 | 0.69 | 0.71 | 0.84 | 0.16 | 0.737 | 0.702 | 0.985 |
| *Paludibacter* | 0.05 | 0.86 | 3.63 | 0.49 | 0.57 | 0.651 | 0.079 | 0.027 |
| *Bacteroides* | 0.74 | 0.39 | 0.15 | 0.27 | 0.12 | 0.968 | 0.215 | 0.433 |
| *Prevotella2* | 0.23 | 1.09 | 0.11 | 0.09 | 1.57 | 0.508 | 0.137 | 0.399 |
| *Parabacteroides* | 0.05 | 0.11 | 0.38 | 0.18 | 0.07 | 0.954 | 0.327 | 0.515 |
| *Butyrivibrio* | 0.05 | 0.43 | 0.22 | 0.09 | 0.07 | 0.623 | 0.975 | 0.246 |
| *Epulopiscium* | 0.02 | 0.15 | 0.12 | 0.06 | 0.04 | 0.728 | 0.804 | 0.459 |
| *Succinivibrio* | 1.83 | 0.61 | 0.11 | 0.29 | 0.23 | 0.952 | 0.035 | 0.199 |

^1^ Main effects of treatment and housing ^2^ Interaction between treatment and housing

**Supplementary Table S2: An assessment of the impact of Vitamin D supplementation on Alpha Diversity in oral samples**

|  | Indoor | | Outdoor | |  |  |  |  |
| --- | --- | --- | --- | --- | --- | --- | --- | --- |
|  | Control | VitD | Control | VitD | SEM | Trt^1^ | Housing^1^ | Trt*Housing^2^ |
| Observed | 154.17 | 127.00 | 116.75 | 84.83 | 37.96 | 0.336 | 0.473 | 0.954 |
| Chao1 | 303.71 | 209.74 | 208.19 | 181.74 | 52.76 | 0.284 | 0.296 | 0.554 |
| Shannon | 4.09 | 3.62 | 3.96 | 3.59 | 0.34 | 0.830 | 0.259 | 0.891 |
| Simpson | 0.95 | 0.93 | 0.96 | 0.94 | 0.03 | 0.643 | 0.407 | 0.907 |
| Fisher | 72.87 | 60.45 | 65.83 | 52.76 | 15.91 | 0.668 | 0.460 | 0.614 |

^1^ Main effects of treatment and housing ^2^ Interaction between treatment and housing

**Supplementary Table S3: Differential abundance analysis of the impact of housing and vitamin D supplementation on the oral microbiome**

|  | Indoor | | Outdoor | |  |  |  |  |
| --- | --- | --- | --- | --- | --- | --- | --- | --- |
|  | Control | VitD | Control | VitD | SEM | Trt^1^ | Housing^1^ | Trt*Housing |
| **Phylum** |  |  |  |  |  |  |  |  |
| Firmicutes | 66.2 | 53.55 | 50.53 | 54.55 | 4.87 | 0.259 | 0.044 | 0.023 |
| Proteobacteria | 10.29 | 20.53 | 29.45 | 31.56 | 4.86 | 0.001 | 0 | 0.006 |
| Fusobacteria | 15.39 | 12.11 | 5.43 | 3.54 | 2.52 | 0.059 | 0 | 0.58 |
| Bacteroidetes | 5.38 | 9.98 | 5.98 | 6 | 0.86 | 0.079 | 0.245 | 0.083 |
| Tenericutes | 0.32 | 0.26 | 3.93 | 1.69 | 0.78 | 0.412 | 0.003 | 0.633 |
| Actinobacteria | 1.8 | 2.34 | 0.61 | 0.36 | 0.28 | 0.776 | 0.007 | 0.42 |
| Cyanobacteria | 0.27 | 0.61 | 4 | 2.02 | 0.82 | 0.915 | 0.002 | 0.184 |
| **Family** |  |  |  |  |  |  |  |  |
| *Ruminococcaceae* | 34.84 | 27.73 | 32.54 | 32.61 | 3.56 | 0.162 | 0.553 | 0.155 |
| *Lachnospiraceae* | 14.24 | 7.69 | 8.65 | 9.58 | 1.49 | 0.087 | 0.339 | 0.021 |
| *Pasteurellaceae* | 2.12 | 15.65 | 24.89 | 29.02 | 5.14 | 0 | 0 | 0 |
| *Fusobacteriaceae* | 7.01 | 7.12 | 4.63 | 3.63 | 1.13 | 0.554 | 0.01 | 0.501 |
| *Neisseriaceae* | 3.88 | 3.68 | 3.6 | 0.87 | 0.93 | 0.023 | 0.02 | 0.033 |
| *Leptotrichiaceae* | 9.95 | 6.35 | 1.39 | 0.74 | 2.03 | 0.106 | 0 | 0.778 |
| *Moraxellaceae* | 2.95 | 0.63 | 3.36 | 2.42 | 0.73 | 0.024 | 0.067 | 0.126 |
| *Peptostreptococcaceae* | 4.24 | 3.22 | 1.89 | 2.98 | 0.57 | 0.73 | 0.101 | 0.17 |
| *Clostridiaceae* | 2.96 | 1.44 | 2.12 | 2.18 | 0.35 | 0.277 | 0.897 | 0.243 |
| *Tissierellaceae* | 2.75 | 2.73 | 1.4 | 1.48 | 0.38 | 0.943 | 0.052 | 0.922 |
| *Flavobacteriaceae* | 1.94 | 4.26 | 1.22 | 4.39 | 0.58 | 0.002 | 0.452 | 0.393 |
| *Mycoplasmataceae* | 0.31 | 0.21 | 4.14 | 1.85 | 0.91 | 0.385 | 0.003 | 0.782 |
| *Weeksellaceae* | 2.31 | 5.53 | 0.47 | 0.8 | 0.55 | 0.106 | 0.001 | 0.686 |
| *Sphingomonadaceae* | 0.59 | 0.42 | 0.89 | 2.1 | 0.09 | 0.632 | 0.076 | 0.276 |
| *Micrococcaceae* | 0.81 | 0.87 | 0.58 | 0.09 | 0.14 | 0.286 | 0.128 | 0.25 |
| *Prevotellaceae* | 1.06 | 0.38 | 0.37 | 0.08 | 0.12 | 0.177 | 0.172 | 0.779 |
| *Succinivibrionaceae* | 0.37 | 0.16 | 1.04 | 0.09 | 0.18 | 0.119 | 0.821 | 0.434 |
| *Mogibacteriaceae* | 0.94 | 0.64 | 0.39 | 0.4 | 0.14 | 0.768 | 0.265 | 0.734 |
| *Porphyromonadaceae* | 0.31 | 0.34 | 0.66 | 0.08 | 0.11 | 0.299 | 0.71 | 0.26 |
| *Rikenellaceae* | 0.13 | 0.13 | 0.91 | 0.33 | 0.16 | 0.608 | 0.162 | 0.623 |
| *Streptococcaceae* | 0.22 | 3.55 | 0.48 | 0.05 | 0.55 | 0.853 | 0.119 | 0.031 |
| *Planococcaceae* | 0.39 | 0.92 | 0.19 | 0.24 | 0.11 | 0.483 | 0.183 | 0.696 |
| *Corynebacteriaceae* | 1.11 | 1.55 | 0.06 | 0.19 | 0.14 | 0.472 | 0.021 | 0.702 |
| *Sphingobacteriaceae* | 0.11 | 0.08 | 0.37 | 0.3 | 0.31 | 0.852 | 0.299 | 0.999 |
| *Staphylococcaceae* | 1.44 | 1.22 | 0.02 | 0.81 | 0.25 | 0.264 | 0.148 | 0.225 |
| *Hyphomicrobiaceae* | 0.12 | 0.38 | 0.16 | 0.09 | 0.08 | 0.779 | 0.62 | 0.455 |
| *Aerococcaceae* | 0.32 | 0.5 | 0.05 | 0.53 | 0.11 | 0.208 | 0.416 | 0.385 |
| *Bacteroidaceae* | 0.04 | 0.13 | 0.32 | 0.05 | 0.75 | 0.831 | 0.731 | 0.343 |
| *Coriobacteriaceae* | 0.1 | 0.17 | 0.08 | 0.14 | 0.05 | 0.661 | 0.85 | 0.98 |
| **Genus** |  |  |  |  |  |  |  |  |
| *Fusobacterium* | 18.19 | 16.35 | 8.99 | 8.45 | 2.56 | 0.509 | 0 | 0.86 |
| *Actinobacillus* | 2.93 | 17.47 | 21.91 | 27.51 | 5.04 | 0 | 0 | 0 |
| *Ruminococcus* | 11.99 | 2.25 | 3.61 | 1.5 | 1.21 | 0 | 0.007 | 0.15 |
| *Moraxella* | 4.54 | 1 | 9.03 | 5.92 | 2.09 | 0.004 | 0.001 | 0.075 |
| *Faecalibacterium* | 6.24 | 3.78 | 3.46 | 3.13 | 1.14 | 0.191 | 0.094 | 0.375 |
| *Clostridium* | 4.63 | 1.68 | 4 | 3.63 | 0.86 | 0.048 | 0.248 | 0.096 |
| *Coprococcus* | 3.56 | 2.14 | 2.79 | 1.95 | 0.48 | 0.136 | 0.549 | 0.792 |
| *Dorea* | 4.6 | 2.09 | 2.12 | 4.01 | 0.66 | 0.778 | 0.812 | 0.014 |
| *Oscillospira* | 2.02 | 2.84 | 4.03 | 6.88 | 1.31 | 0.089 | 0.005 | 0.694 |
| *Ornithobacterium* | 4.41 | 7.51 | 1.16 | 1.04 | 0.85 | 0.497 | 0 | 0.31 |
| *Prevotella1* | 2.36 | 0.89 | 1.14 | 0.39 | 0.30 | 0.046 | 0.123 | 0.922 |
| *Blautia* | 2.42 | 1.68 | 1.02 | 1.01 | 0.34 | 0.622 | 0.08 | 0.628 |
| *Helcococcus* | 1.33 | 2.76 | 1.82 | 2.88 | 0.62 | 0.065 | 0.561 | 0.659 |
| *Succinivibrio* | 0.88 | 0.31 | 2.39 | 0.23 | 0.38 | 0.021 | 0.607 | 0.348 |
| *Sphingomonas* | 0.91 | 0.89 | 2.3 | 4.7 | 0.66 | 0.372 | 0.003 | 0.345 |
| *Parvimonas* | 1.98 | 0.4 | 0.56 | 0.23 | 0.42 | 0.075 | 0.185 | 0.59 |
| *Streptococcus* | 0.73 | 6.87 | 1.1 | 0.11 | 1.06 | 0.988 | 0.015 | 0.004 |
| *Capnocytophaga* | 0.64 | 0.5 | 1.08 | 1.43 | 0.33 | 0.98 | 0.139 | 0.606 |
| *Peptostreptococcus* | 0.3 | 1.04 | 0.87 | 0.27 | 0.27 | 0.95 | 0.829 | 0.072 |
| *Butyrivibrio* | 0.72 | 0.54 | 0.26 | 0.5 | 0.14 | 0.77 | 0.406 | 0.47 |
| *Epulopiscium* | 0.37 | 0.12 | 0.45 | 1.04 | 0.26 | 0.849 | 0.193 | 0.273 |
| *Peptoniphilus* | 0.37 | 0.89 | 0.34 | 0.11 | 0.15 | 0.991 | 0.214 | 0.241 |
| *GW-34* | 0.67 | 0.85 | 0.16 | 0.78 | 0.24 | 0.194 | 0.276 | 0.337 |
| *Mogibacterium* | 0.48 | 0.86 | 0.23 | 0.5 | 0.16 | 0.306 | 0.339 | 0.884 |
| *Devosia* | 0.26 | 0.78 | 0.27 | 0.2 | 0.15 | 0.61 | 0.41 | 0.376 |
| *5-7N15* | 0.08 | 0.28 | 0.74 | 0.11 | 0.16 | 0.784 | 0.542 | 0.164 |
| *Gallicola* | 0.35 | 0.23 | 0.16 | 0.23 | 0.12 | 0.964 | 0.673 | 0.679 |
| *Staphylococcus* | 2.48 | 1.94 | 0.02 | 0.91 | 0.43 | 0.236 | 0.07 | 0.179 |
| *Paludibacter* | 0.05 | 0.17 | 0.91 | 0.39 | 0.19 | 0.895 | 0.125 | 0.394 |
| *Flavobacterium* | 0.05 | 0.95 | 0.61 | 6.64 | 1.26 | 0.014 | 0.036 | 0.794 |
| *Anaerostipes* | 0.07 | 0.35 | 0.34 | 0.39 | 0.13 | 0.393 | 0.413 | 0.475 |
| *Aequorivita* | 0.27 | 2.06 | 0.06 | 0.2 | 0.20 | 0.138 | 0.082 | 0.657 |
| *Jeotgalicoccus* | 0.67 | 0.56 | 0.02 | 1.04 | 0.27 | 0.222 | 0.349 | 0.185 |
| *Chryseobacterium* | 0.19 | 0.12 | 0.02 | 0.71 | 0.13 | 0.379 | 0.912 | 0.246 |

^1^ Main effects of treatment and housing ^2^ Interaction between treatment and housing

**Supplementary Table S4: Compositional analysis of (a) milk replacer and (b) calf pellets fed to calves**

| 1. **Complete Milk Replacer Feed for Calves*** | |
| --- | --- |
|  |  |
| **Analytical Constituents** |  |
| Crude Protein | 23.0% |
| Crude Fibre | Nil |
| Crude Oils and Fats | 18.0% |
| Crude Ash | 7.5% |
| Calcium | 0.8% |
| Sodium | 0.5% |
| Phosphorus | 0.7% |
|  |  |
|  |  |
| **Composition** |  |
| Whey protein |  |
| Vegetable Oil (Palm & Coconut) |  |
| Hydrolysed Wheat Gluten |  |
| Calcium Carbonate |  |
| Magnesium Oxide |  |
| Gardion (Garlic) at 225 mg per kg |  |
| Genial Dosto Oregano (Flavouring Compound) at 4000 mg per kg |  |
|  |  |
| **Additives** | **per kg** |
| Vitamin A | 25,000 IU |
| Vitamin D3 | 6,000 IU |
| Vitamin E | 250 IU |
| Copper | 10 mg |
| Iodine | 0.25 mg |
| Iron | 80 mg |
| Manganese | 30 mg |
| Selenium | 0.4 mg |
| Zinc | 50 mg |
| BHT (antioxidant) | 150 mg |
| Citric acid | 1000 mg |
|  |  |
| *Volac Blossom EasyMix |  |

| **(b) Calf Pellet*** |  |
| --- | --- |
|  |  |
| **Analytical Constituents** |  |
|  |  |
| Crude Protein | 18.8% |
| Crude Fibre | 8.0% |
| Crude Oil & Fats | 3.3% |
| Crude Ash | 7.1% |
| Sodium | 0.3% |
|  |  |
| **Composition** |  |
| Barley |  |
| Soya (Bean) |  |
| Meal Dehulled |  |
| Maize |  |
| Dried Beet Pulp Molassed |  |
| Soya (Bean) Hulls |  |
| Cane Molasses |  |
| Calcium carbonate |  |
| Sodium chloride |  |
| Monocalcium phosphate |  |
| Vegetable oil blend |  |
|  |  |
| **Additives** | **per kg** |
| Vitamin A | 10,000 IU |
| Vitamin D3 | 2,000 IU |
| Vitamin E | 40 mg |
| Iodine | 8 mg |
| Cobalt | 40 mg |
| Cupric chelate | 48 mg |
| Cupric sulphate pentahydrate | 40 mg |
| Manganese | 81 mg |
| Zinc Oxide | 139 mg |
| Selenium | 11 mg |
|  |  |
| *Lakeland Agri Ltd. |  |
|  |  |
